# Characterization of point-spread function specification error on Geometric Transfer Matrix partial volume correction in [^11^C]PiB amyloid imaging

**DOI:** 10.1186/s40658-021-00403-5

**Published:** 2021-07-20

**Authors:** Charles M. Laymon, Davneet S. Minhas, Sarah K. Royse, Howard J. Aizenstein, Ann D. Cohen, Dana L. Tudorascu, William E. Klunk

**Affiliations:** 1 Department of Radiology, University of Pittsburgh, Pittsburgh, PA USA; 2 Department of Psychiatry, University of Pittsburgh, Pittsburgh, PA USA; 3 Department of Bioengineering, University of Pittsburgh, PET Center, PUH B930, 200 Lothrop St, Pittsburgh, PA 15213 USA

**Keywords:** Nuclear imaging, Brain, Evaluation and performance, Quantification and estimation, Positron emission tomography, Partial volume effect correction

## Abstract

**Purpose:**

Partial-volume correction (PVC) using the Geometric Transfer Matrix (GTM) method is used in positron emission tomography (PET) to compensate for the effects of spatial resolution on quantitation. We evaluate the effect of misspecification of scanner point-spread function (PSF) on GTM results in amyloid imaging, including the effect on amyloid status classification (positive or negative).

**Methods:**

Twenty-nine subjects with Pittsburgh Compound B ([^11^C]PiB) PET and structural T1 MR imaging were analyzed. FreeSurfer 5.3 (FS) was used to parcellate MR images into regions-of-interest (ROIs) that were used to extract radioactivity concentration values from the PET images. GTM PVC was performed using our “standard” PSF parameterization [3D Gaussian, full-width at half-maximum (*w*) of approximately 5 mm]. Additional GTM PVC was performed with “incorrect” parameterizations, taken around the correct value. The result is a set of regional activity values for each of the GTM applications. For each case, activity values from various ROIs were combined and normalized to produce standardized uptake value ratios (SUVRs) for nine standard [^11^C]PiB quantitation ROIs and a global region. GTM operating-point characteristics were determined from the slope of apparent SUVR versus *w* curves.

**Results:**

Errors in specification of *w* on the order of 1 mm (3D) mainly produce only modest errors of up to a few percent. An exception was the anterior ventral striatum in which fractional errors of up to 0.29 per millimeter (3D) of error in *w* were observed.

**Conclusion:**

While this study does not address all the issues regarding the quantitative strengths and weakness of GTM PVC, we find that with reasonable caution, the unavoidable inaccuracies associated with PSF specification do not preclude its use in amyloid quantitation.

**Supplementary Information:**

The online version contains supplementary material available at 10.1186/s40658-021-00403-5.

## Background

Image-based partial volume correction is frequently used in positron emission tomography (PET) to compensate for the effects of imperfect spatial resolution on image quantitation. A commonly employed procedure is the Geometric Transfer Matrix (GTM) method [1], which is essentially a deconvolution process with an implicitly assumed noise model [2]. The procedure begins with the assumption that the true distribution of activity is in the form of a set of predefined regions spanning the brain, each with a homogeneous concentration of activity. The method also assumes that the true distribution of activity is related to the measured distribution via an image-based convolution operation, and is usually implemented assuming a translationally invariant convolution kernel with a simple functional form.

While both assumptions are important, this work specifically examines the effects of convolution kernel misspecification. The convolution kernel is frequently taken to be Gaussian with a full-width at half-maximum (*w*) determined from point-source measurements. However, the true functional form of the scanner point-spread function (PSF) is not Gaussian and is not translationally invariant [3]. Additionally, point-source measurements made with one positron emitting isotope are not entirely appropriate for use with tracers labeled with a different isotope. For example, the mean positron range in water for ^18^F is 0.6 mm while this value is 1.2 mm for ^11^C and 3.0 mm ^15^O [4].

Several authors [5–10] have reported on resolution measurements of the 4-ring mCT PET/CT using NEMA 2007 or 2012 standards, which call for measurements to be made at several specified positions within the PET field of view (FOV). All work cited yielded similar results to those of Rausch et al. [10], who, using the NEMA 2012 protocol found *w* values of 4.25 mm, 5.85 mm, and 7.80 mm at radial offsets of 1 cm, 10 cm, and 20 cm respectively, all measured in the axial direction. Transaxially, *w* values were 4.33 mm (transverse) at a radial offset of 1 cm, 5.16 (transverse radial) and 4.72 (transverse tangential) at 10 cm radial offset, and 5.55 (transverse radial) and 6.48 (transverse tangential) at 20 cm radial offset. Each of these values is an average over data acquired at the axial center of the PET FOV and at a distance of 3/8 of the axial FOV from the center, which, for the mCT, corresponds to approximately 8 cm. We note that all measurements were performed using point sources in air as is consistent with NEMA protocols.

In our standard analysis procedures, for the purposes of applying an image-based partial volume correction (PVC), we simplistically model our 4-ring mCT scanner’s point-spread function as a translationally invariant Gaussian with a nearly isotropic *w* value of 5 mm (transverse, radial, and tangential) and 4.8 mm (axial). These values were based on averages of measurements using ^18^F point sources in water with no background activity. Our use of a water-filled container (unlike the NEMA protocol) was to estimate the system PSF using a setup that more closely models the full imaging situation than does the NEMA protocol which is more aimed at characterizing direct scanner effects.

In amyloid PET imaging, GTM PVC results in substantial modification of outcome values, including standardized uptake value ratios (SUVR), which in turn, can affect amyloid-status classification, diagnostic group effect size, and longitudinal change measures [11–16]. Given the large effect of PVC, it is prudent to understand the functional characteristics of the procedure, including the consequences of unavoidable inaccuracies in the parameterization of the scanner PSF. Others have recognized a similar need in neuro imaging with other tracers and have investigated some aspects of the problem. In their development of a partial-volume correction tool box for neuro imaging, Thomas et al. [17] examined the effects of using an assumed PSF kernel that is mismatched to the actual kernel for the case of FDG imaging. Similarly, the effect of inaccurate PSF specification was partially addressed in the work of Oyama et al. [18] who evaluated several partial-volume correction methods for the tau tracer [^18^F]THK-5351. Both of these studies used simulated data.

The goal of the present study is to perform an in-depth evaluation of the sensitivity of the GTM method to scanner point-spread function specification in [^11^C]PiB imaging using real scan data. We first aim to map the dependence of GTM PVC on regional uncorrected SUVR and assumed PSF *w*. We then aim to characterize GTM PVC errors that could reasonably be expected due to PSF misspecification. One particular concern addressed in this work is the potential for misclassification of the amyloid status (positive or negative) of subjects due to such errors.

## Methods

### Subjects

Scans from subjects who previously had PET and magnetic resonance (MR) imaging acquired and analyzed at the University of Pittsburgh were selected for this analysis. Because this cohort is being used in a separate analysis, subjects must also have had a scan with the tau tracer, [^18^F]AV-1451. Scans were selected based on previously determined uncorrected SUVRs (reference region: cerebellar gray matter) as described in the Image Processing and [^11^C]PiB Quantitation sections below, with the goal of spanning the range of observed [^11^C]PiB SUVR in our facility. To avoid inclusion of too many scans at the low end of [^11^C]PiB SUVR uptake spectrum, the final selection was limited to 29 subjects (74.4 ± 5.3 years, 17F/12M).

Selected PET scans had global [^11^C]Pittsburgh Compound-B ([^11^C]PiB) SUVRs spanning the range of 0.97-2.42. Of these, 15 were classified as globally [^11^C]PiB positive and 14 were classified as globally [^11^C]PiB negative using our current standard threshold for non-partial-volume-corrected data of 1.346 developed from a sparse k-means-analysis modified [19] from the work of Cohen et al. [20]. All studies used in this analysis were performed under protocols approved by the University of Pittsburgh Institutional Review Board. All subjects provided written informed consent.

### Image acquisition

All subjects received T1-weighted MR scans on a Siemens PRISMA 3T scanner using a sagittal Magnetization Prepared Rapid Gradient Echo (MPRAGE) sequence (TE = 2.22 ms, TR = 2400 ms, flip angle = 8 deg).

PET scans were acquired using a Siemens mCT PET/CT scanner. Subjects were injected with 560 MBq (nominal) of [^11^C]PiB 50 min prior to the start of PET component of the scans. Prior to the PET, a low dose CT was acquired without contrast for the purpose of attenuation and scatter correction of the PET data. PET emission data were acquired over the interval 50-70 min post-injection. To allow for investigation of possible subject motion during the scan, raw PET data (sinograms) were binned into 4 5-min frames that were then reconstructed by FORE/Filtered back projection. In keeping with our quantitation pipeline, no post-reconstruction filtering was applied. PET reconstruction was performed using the manufacturer’s software and included corrections for scatter, deadtime, random coincidences, and decay.

### Image processing

PET images were evaluated for frame-to-frame subject motion. For each scan, a set of frames (from 1 frame to all frames) in which no motion was visually apparent was identified and averaged. A set of contours was produced capturing details of the averaged image. All individual frames were evaluated for translational or rotational displacements with reference to the contour set. If such motion was visually apparent, the average image was recreated excluding these frames. If the final averaged image was produced using all frames, i.e., if no frame-to-frame motion was detected in this process, then no motion correction steps were applied. Otherwise each individual frame was registered to the average image and the final set of registered images was then averaged to produce the final single-frame, motion-corrected image representing average tracer uptake over the full 50-70-min acquisition time period. In the current study, 4 of the 29 scans required the motion-correction registration step. A final PET image was produced by rigidly registering the PET to the MR. Image registration for the purposes of motion correction and for matching the PET image to the MR was performed via PMOD 3.709 (PMOD Technologies, Zurich, Switzerland) using normalized mutual information and tri-linear interpolation.

FreeSurfer version 5.3 (FS) was used to parcellate each subject’s native-space MR image into a full set of regions-of-interest (ROIs) spanning the brain. In addition, the Imperial College London Clinical Imaging Centre (CIC) atlas [21] was applied to the native-space MRI to replace default FS atlas striatum ROIs, as previously described [22]. FreeSurfer MRI segmentation resulted in between 190 and 194 FS ROIs for each of the 29 subjects where the difference in number is due to optional regions, e.g., some ventricles that FS identifies in some subjects but not others. The generated FS ROIs were used to extract radioactivity concentration values from the 50-70-min registered PET images.

### GTM partial volume correction

Partial volume correction was performed using the GTM method [1], implemented in Matlab. The code was validated against the GTM feature available within PMOD. In all applications of GTM, the starting parcellations and associated regional activities were those determined as described above. To map out the effects of assumed PSF width, GTM PVC was performed with the “Standard” *w* values (5.0 mm × 5.0 mm × 4.8 mm), used with our mCT, and repeated with Standard ± 0.5 mm (5.0 ± 0.5 mm × 5.0 ± 0.5 mm × 4.80 ± 0.5 mm), Standard ± 1.0 mm, and Standard ± 3.0 mm *w* values. To gauge the effects of performing a minimal PVC, an additional GTM PVC was performed using a *w* value of 0.5 mm uniformly in all directions. The result of these procedures is a set of regional activity values for the FS ROIs for each of the 8 GTM applications as well as a set of values for the case of no GTM correction (*w* = 0).

### [^11^C]PiB quantitation

For each GTM case, FS regional activity values (after GTM correction) from various FS ROIs were combined (volume-weighted average) to produce radioactivity concentration values for a standard set of nine [^11^C]PiB ROIs (anterior cingulate, anterior ventral striatum, superior frontal, orbital frontal, insula, lateral temporal, parietal, posterior cingulate, and precuneus) and a combined global region. Forty-six of the FS ROIs are used in the process, the combinations of which are delineated in Supplemental Table 1. Cerebellar gray matter radioactivity values were used to normalize [^11^C]PiB ROI values to generate regional SUVR measures. Normalization by cerebellum gray matter activity was performed after each GTM correction.

### Geometric transfer matrix evaluation

Starting with the various versions of the GTM-corrected regional [^11^C]PiB activity values, the overall functional characteristics of GTM were determined by examining the apparent [^11^C]PiB SUVR as a function of the assumed point-spread function full-width at half-maximum for each of the standard quantitation regions described above.

We defined GTM raw activity correction factors for the [^11^C]PiB quantitation regions as

$$ {C}_{Raw}(w)=\frac{a(w)}{a\left(w=0\right)}, $$

where *a*(*w*) is the apparent raw radioactivity, without cerebellar normalization after GTM PVC using a full-width at half-maximum of *w*, and *a*(*w* = 0) refers to the uncorrected measured concentration. The quantity *C*_*Cer*_(*w*) is used to represent the raw correction factor specifically for the cerebellar gray matter.

We defined an SUVR correction factor similarly as:

$$ {C}_{SUVR}(w)=\frac{SUVR(w)}{SUVR\left(w=0\right)} $$

where *SUVR*(*w*) represents the apparent SUVR (i.e., raw GTM-corrected activity normalized by cerebellum GTM-corrected activity) using an assumed full-width at half-maximum of *w*.

Of particular interest are the characteristics of the GTM correction in the vicinity of *w*_0_, the value of *w* used in routine application of GTM (the operating point). In our case, *w*_0_ = 5 mm. We examined GTM characteristics at the operating point by estimating the derivative of *C*_*X*_ (where *X* represents Raw, Cer, or SUVR) at *w*_0_ as:

$$ {\left.\frac{d{C}_X}{dw}\right|}_{w_0}=\frac{\left\{{C}_X\left(w=5.5\ \mathrm{mm}\right)-{C}_X\left(w=4.5\ \mathrm{mm}\right)\right\}}{1\ \mathrm{mm}} $$

Results are presented in terms of a fractional error (*FE*_*X*_) per millimeter of error in the assumed value of *w*, defined as

$$ F{E}_X={\left.\frac{d{C}_X/ dw}{C_X}\right|}_{w_0} $$

For the calculation of SUVR fractional error, it is straightforward to show that

1 $$ F{E}_{SUVR}=F{E}_{Raw}-F{E}_{Cer} $$

where the minus sign in Eq. (1) arises because of the division operation used in SUVR normalization.

The behavior of the reference region in response to the various GTM corrections was directly examined by converting raw cerebellum gray matter activity to SUV (units of gm/ml), where SUV is calculated by dividing the raw activity concentration, decay corrected to injection time, by injected dose and multiplying by subject body mass.

### Statistics

Descriptive statistics were calculated using Matlab. The mean, root mean square, standard deviation of FE values for each quantitation region across subjects were calculated using the mean, rms, sd, min, and max functions of Matlab respectively. Linear fits of FE versus SUVR correction factor *C*_*SUVR*_ were produced using the Matlab polyfit function.

Separately, FE was fitted to a constant + linear + asymptotic function, phenomenologically found to provide a reasonable fit to the data, of the form:

2 $$ y={b}_0+{b}_1x+{b}_2\left[1-\exp \left(-{b}_3x\right)\right] $$

where the predictor variable *x* is GTM-corrected SUVR, the response variable *y* is FE and each subscripted *b* is a fit parameter. The Matlab function fitnlm was used for this procedure with default settings. The purpose of producing the fits was mainly to allow estimates of the correction-factor values associated with an FE of 0 in the first case and, in the second case, to estimate the FE value in the vicinity of the University of Pittsburgh amyloid positivity SUVR thresholds, determined is a sparse k-means analysis of a separate set of subjects [19, 20].

## Results

### Overall characterization of GTM as a function of PVC full-width at half-maximum

**Fig. 1:**
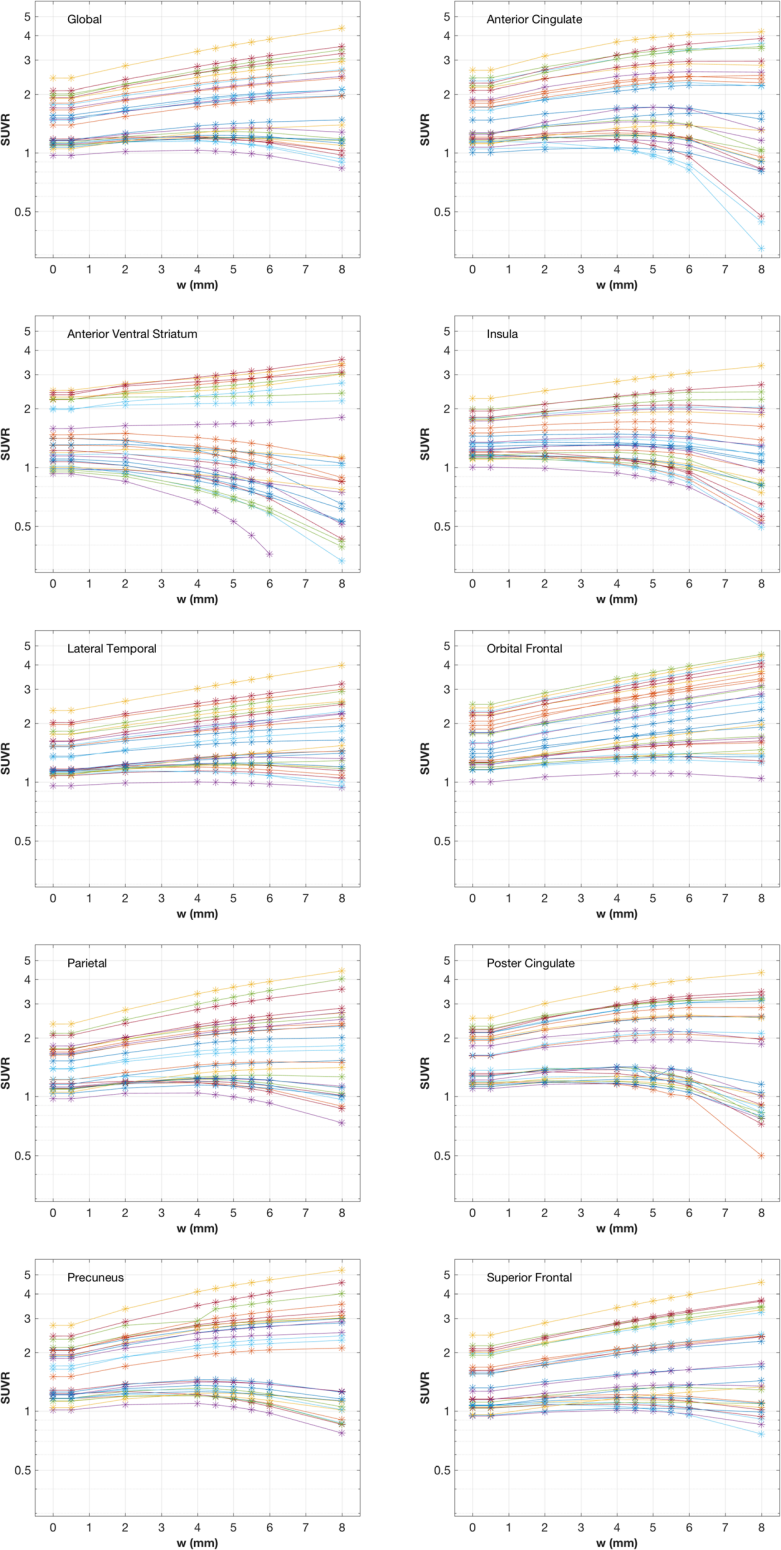
Plots of apparent SUVR vs the full-width at half-maximum (*w*) of the Gaussian kernel used in the applied GTM correction. The SUVR scale is logarithmic. Plots are shown for the global region and all [^11^C]PiB quantitation regions. The *w* values indicated are the values applied in the *x* and *y* directions (transaxially) and nearly so in the axial direction (see text). Separate colors are used for each of the 29 subjects and span the range of typical [^11^C]PiB uptake. Individual measurements are shown by plot symbols; lines are shown only as a guide. Points at *w* = 0 correspond to results with no GTM applied whereas those at *w* = 5 correspond to application of GTM PVC using our standard Gaussian kernel

The main results of this work are shown in Fig. 1 in which SUVR is plotted for the global [^11^C]PiB region and constituent [^11^C]PiB ROIs as a function of *w* used in performing a GTM PVC. The plots illustrate the magnitude of the effect of GTM PVC on SUVR. Compared to uncorrected values (*w* = 0), the application of the GTM with the standard kernel (*w* = 5) spreads out the SUVR measures for all regions, a finding that is consistent with reports of PVC-associated effect size increases (i.e., separation of [^11^C]PiB- and [^11^C]PiB+ subjects) [12]. We observe that all trajectories have 0 slope at *w* = 0, a finding consistent with expectations. See Additional file 3 (Supplemental Material) for a discussion of the GTM properties in the vicinity of *w* = 0. The effect of GTM and GTM specification error on the cerebellar gray matter reference region was small compared to the overall effect on SUVR (see Supplemental Figure 1).

Trajectory shapes are relatively simple, ranging from monotonically increasing to monotonically decreasing with many rising at low *w* values, reaching a broad maximum, and then falling off at higher *w* values. Further, there is minimal crossing of curves so that trajectories are reasonably distinguishable. We observe that every region has a set of trajectories with approximately 0 slope at our operating point (*w* = 5 mm). For example, in the global region, we find that trajectories with an SUVR of about 1.2 at a *w* of 5 mm have a slope of about 0 at that point.

### Operating point characteristics

**Fig. 2:**
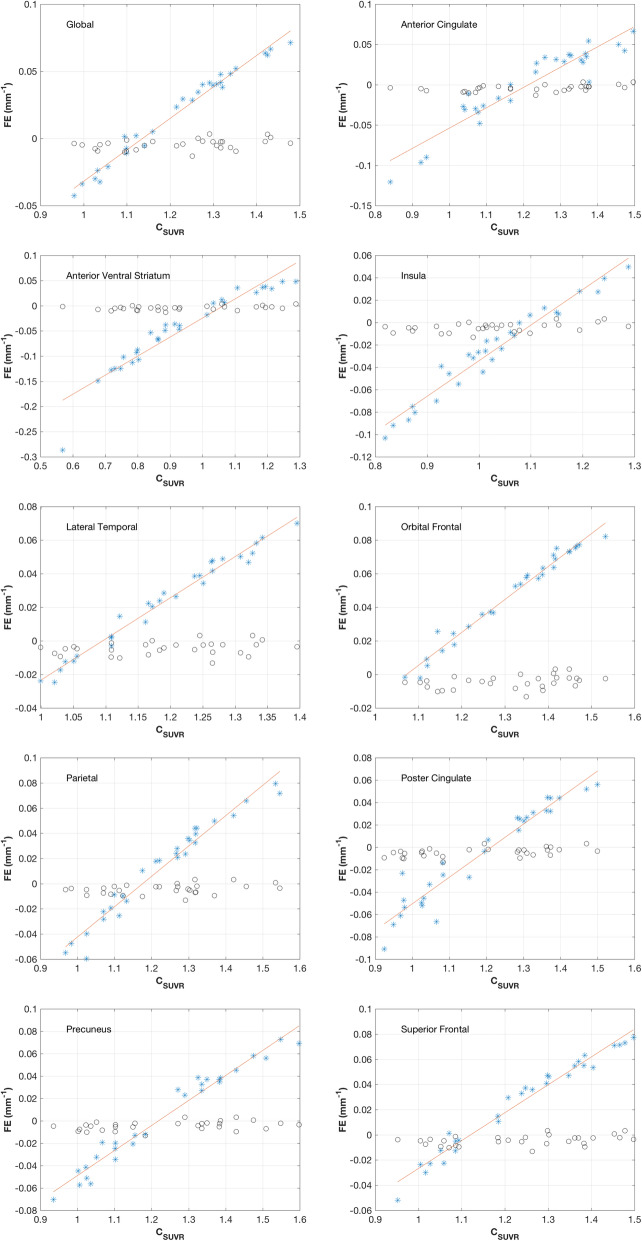
GTM operating point characteristics showing fractional error (*FE*) in GTM correction factor per millimeter of PSF w-error in the vicinity of our operating point (*w* = *w*_0_ = 5 mm) plotted as a function of GTM SUVR correction factor (*C*_*SUVR*_). Each point in a plot derives from an individual subject. The blue points show the SUVR fractional error (*FE*_*SUVR*_) and the line is a best fit. The black circles show the cerebellum fractional error (*FE*_*Cer*_) and illustrate the (negative) contribution of the cerebellum to the fractional SUVR error. Both fractional errors are presented as a function of the regional SUVR correction factor. Thus, there are corresponding black and blue points at each *C*_*SUVR*_ value

In Fig. 2, *FE*_*SUVR*_, the fractional SUVR error per millimeter error in *w* at the operating point (*w* = 5 mm), is plotted against the SUVR GTM factor at the operating point for all [^11^C]PiB quantification regions and for the global region. We find an approximately linear relationship between these quantities for the global and the individual regions, as shown by the best-fit lines. Results for all regions are provided in Table 1 that lists, for each region, the coefficient of determination (*R*^2^) of the linear fit, and the slope and *y*-intercept of the fit. We also find that for each region, the fit lines cross the *x*-axis at some point over the range of *C*_*SUVR*_ values encountered in this study. These are listed in Table 1 under the *C*_*SUVR*_-intercept heading. The figures also show the values of *FE*_*Cer*_, the cerebellar gray matter (negative) contribution to the SUVR fractional error. Cerebellum activity correction factors *C*_*Cer*_(*w* = 5mm) (not shown) span a tight range, compared to the *C*_*SUVR*_, with values from 1.02 to 1.11, a mean of 1.06 and a standard deviation of 0.02.

**Table 1:** Parameters of the linear fits to the data shown in Fig. 2

Region	***R***^**2**^	Slope (mm^**−1**^ SUVR^**−1**^)	FE-intercept (mm^**−1**^)	C-intercept (SUVR)
Global	0.978	0.234	−0.266	1.136
Anterior cingulate	0.881	0.252	−0.306	1.214
Anterior ventral striatum	0.896	0.378	−0.403	1.065
Insula	0.954	0.318	−0.352	1.107
Lateral temporal	0.978	0.245	−0.268	1.095
Orbital frontal	0.976	0.196	−0.210	1.073
Parietal	0.958	0.241	−0.283	1.176
Poster cingulate	0.924	0.237	−0.288	1.213
Precuneus	0.965	0.224	−0.272	1.219
Superior frontal	0.970	0.221	−0.248	1.121

An overall error-scale summary is presented in Table 2 that shows mean *FE*_*SUVR*_, root mean square (RMS) of *FE*_*SUVR*_, and the *FE*_*SUVR*_ population standard deviation for each region taken over all subjects. The same quantities are then shown stratified by regional amyloid positivity based on the GTM-corrected SUVR using our standard [^11^C]PiB thresholds. The table also shows the volume of each region, averaged over subjects, as determined in the FreeSurfer analysis.

**Table 2:** SUVR fractional error statistics (mm^−1^) shown for all subjects and for regionally [^11^C]PiB positive and regionally [^11^C]PiB negative subjects separately. Regional volumes averaged over subjects are shown in the last column

	All subjects (***N*** = 29)					Regional [^**11**^C]PiB positive subjects				Regional [^**11**^C]PiB negative subjects				Regional volume (cm^**3**^)
Region	Mean	RMS	SD	Min	Max	N	Mean	RMS	SD	N	Mean	RMS	SD	
Global	0.0196	0.039	0.034	−0.043	0.071	14	−0.0106	0.023	0.021	15	0.0478	0.049	0.013	286.46
Anterior cingulate	0.0003	0.046	0.047	−0.121	0.066	16	−0.0315	0.050	0.040	13	0.0394	0.041	0.011	7.55
Anterior ventral striatum	−0.0497	0.090	0.076	−0.287	0.048	19	-0.0911	0.109	0.061	10	0.0289	0.033	0.016	1.70
Insula	−0.0253	0.047	0.040	−0.103	0.050	16	−0.0531	0.060	0.028	13	0.0090	0.023	0.022	12.77
Lateral temporal	0.0236	0.036	0.028	−0.025	0.070	15	0.0028	0.020	0.020	14	0.0459	0.048	0.013	61.42
Orbital frontal	0.0472	0.054	0.027	−0.002	0.082	13	0.0222	0.028	0.017	16	0.0676	0.068	0.009	21.27
Parietal	0.0125	0.040	0.039	−0.060	0.079	17	−0.0127	0.030	0.028	12	0.0482	0.051	0.016	62.81
Poster cingulate	−0.0070	0.043	0.044	−0.091	0.056	14	−0.0470	0.051	0.021	15	0.0304	0.034	0.016	9.59
Precuneus	0.0050	0.043	0.043	−0.070	0.073	14	−0.0356	0.040	0.018	15	0.0429	0.045	0.015	16.45
Superior frontal	0.0253	0.044	0.037	−0.052	0.077	16	−0.0015	0.026	0.026	13	0.0584	0.059	0.012	92.90

**Fig. 3:**
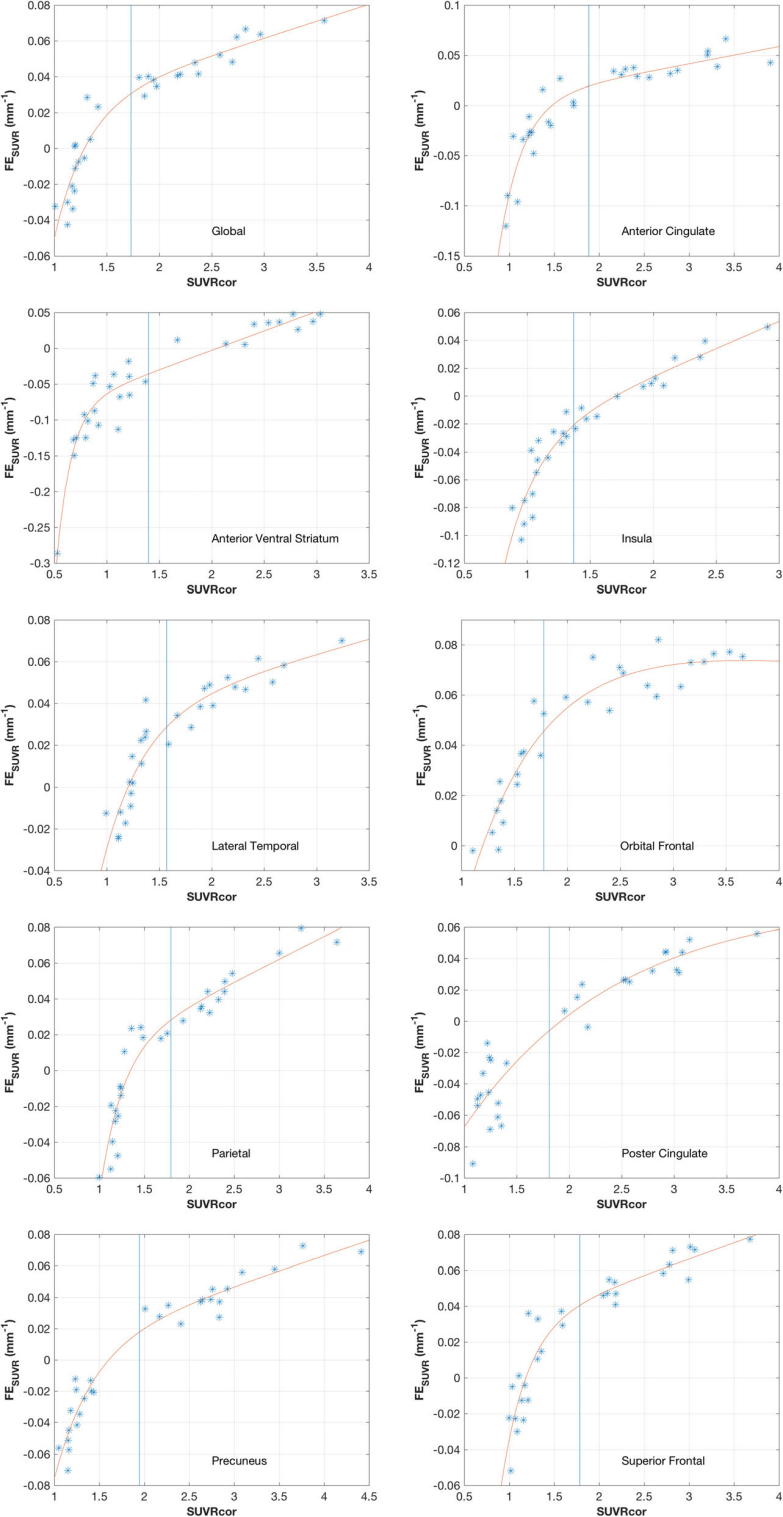
GTM operating point characteristics showing GTM fractional error plotted as a function of GTM-corrected [^11^C]PiB SUVR. A fit to the data, described in the text (equation 2), is included to highlight the trend of the data. Vertical lines show the University of Pittsburgh thresholds for amyloid positivity for GTM-corrected data for each region

Figure 3 presents the fractional SUVR error, *FE*_*SUVR*_, for all regions as in Fig. 2, but plotted as a function of GTM-corrected SUVR using our standard kernel. The curves on the plot are the results of the fits to equation (2). Fits are included mainly to provide a method for deducing an approximate value of *FE*_*SUVR*_ in the vicinity of the [^11^C]PiB (GTM corrected) positivity thresholds indicated by the vertical lines in the plots. Thus, for example, in the global region, in the vicinity of our positivity threshold, we find about a 0.031 GTM-corrected-SUVR fractional error per millimeter of error in the PSF specification. Table 3 summarizes features of Fig. 3 for each region and lists the point at which the fit intercepts the corrected SUVR axis (*x*-axis), the positivity threshold indicated by the vertical lines, and the *FE*_*SUVR*_ at the GTM-corrected SUVR positivity threshold based on the fit.

**Table 3:** Summary of the features of the Fig. 3 fits

Region	***R***^**2**^	SUVR intercept (SUVR)	Positivity threshold (SUVR)	FE at threshold (mm^**−1**^)
Global	0.92	1.29	1.73	0.031
Anterior cingulate	0.88	1.48	1.89	0.019
Anterior ventral striatum	0.92	2.05	1.40	−0.036
Insula	0.92	1.70	1.37	−0.021
Lateral temporal	0.88	1.20	1.57	0.029
Orbital frontal	0.91	1.20	1.78	0.045
Parietal	0.92	1.35	1.80	0.028
Poster cingulate	0.88	1.92	1.81	−0.006
Precuneus	0.95	1.60	1.95	0.018
Superior frontal	0.89	1.18	1.78	0.040

## Discussion

We find that the GTM procedure is well behaved in that trajectories of apparent SUVR as a function of assumed PSF full-width at half-maximum are smooth without sharp excursions. In general, the use of GTM correction causes SUVR to spread over a wider range that depends on the assumed PSF *w* value. Thus, a positive error in the *w* specification would tend to make increasing SUVR values in a longitudinal study appear to increase slightly faster than in the case of no PSF error. Similarly, a negative error would result in an apparent slightly slower rate. The GTM method, assuming valid a priori assumptions and a correct PSF model, is expected to be unbiased for most situations [2]. Thus, it is reasonable to compare the effects of PSF misspecification to the case of not applying GTM correction at all, which is equivalent to applying GTM using a 0-mm *w* value (i.e., in our case, a −5-mm 3D error). This typically results (Fig. 1) in trajectory effects that are larger than those resulting from small PSF specification errors in the neighborhood of the correct *w*.

The operating point analysis shows that misspecification of the PSF produced a relatively modest effect on GTM partial volume correction in most cases. The highest *FE*_*SUVR*_ observed for the global region was 0.071 mm^−1^ (Fig. 2). However, some regions exhibited a substantially greater *FE*_*SUVR*_ for some subjects. The greatest absolute value of *FE*_*SUVR*_ (−0.29 mm^−1^) found in this study occurred in the anterior ventral striatum and a total of 8 subjects had anterior ventral striatum |*FE*_*SUVR*_| values *>* 0.1 mm^−1^. All such values were negative (i.e., *FE*_*SUVR*_ *<* −0.1 mm^−1^) and are associated with subjects who were [^11^C]PiB negative and at the lowest end of the SUVR scale (*<* 1.11, standard GTM corrected). There were two other regions in which |*FE*_*SUVR*_| values *>* 0.1 mm^−1^ were observed. The anterior cingulate yielded one point with an *FE*_*SUVR*_ value of −0.12 mm^−1^ and the insula yielded one point at −0.10 mm^−1^. Both of these are from the same subject and are associated with GTM-corrected SUVR that are well below the corresponding regional positivity thresholds. The largest positive individual *FE*_*SUVR*_ was observed in the orbital frontal region of a [^11^C]PiB positive subject with a value of 0.082 mm^−1^.

The RMS *FE*_*SUVR*_ values listed in Table 2 provide a means for examining error sizes in each of the regions averaged over subjects and accounting for positive and negative values in a single measure. By this method, we again find the anterior ventral striatum to be most sensitive to point-spread-specification error with an RMS *FE*_*SUVR*_ value of 0.090 mm^−1^ and the lateral temporal region to be the least sensitive with an RMS *FE*_*SUVR*_ value of 0.036 mm^−1^. The global region was found to be relatively insensitive to point-spread-specification error with an RMS *FE*_*SUVR*_ value of 0.039 mm^−1^.

Regional sensitivity to PSF specification depends on differences in concentrations between neighboring regions coupled with region size. Our results are consistent with this: while the lateral temporal region is one of the largest regions (average volume: 61 cm^3^) used in this analysis, the anterior ventral striatum, which showed, by far, the greatest sensitivity to PSF specification error was, by far, the smallest, with an average volume of 1.7 cm^3^. As described above and as can be seen in Fig. 2, the relatively large RMS *FE*_*SUVR*_ value of the anterior ventral striatum is driven by subjects with negative *FE*_*SUVR*_ values. These correspond to subjects with low SUVR (Fig. 2). The intended effect of partial volume correction is to undo the blurring inherent in PET imaging. Thus, for most geometries, the correction process boosts contrast between regions. The use of a PSF that is larger than the true value can be thought of as producing an over correction resulting in regional contrast that is too large. Like other amyloid tracers, [^11^C]PiB exhibits nonspecific white matter uptake that is higher than target-region uptake in low-amyloid subjects. Thus, in such subjects, for target regions with large tracts of bordering white matter, the use of too large, a PSF tends to produce too-low values in the target region, i.e., a negative value of *FE*_*SUVR*_. Such effects are particularly apparent in small regions (e.g., anterior ventral striatum) that typically have small volume-to-boundary ratios compared to larger regions.

In addition to the various regional *FE*_*SUVR*_, Fig. 2 also shows *FE*_*Cer*_, the (negative) contribution to *FE*_*SUVR*_ from the GTM correction applied to cerebellar gray matter, which is used to normalize regional activity. In most cases, the cerebellar contribution is small; the exception being, unsurprisingly, when *FE*_*SUVR*_ values are near 0.

For every region, there is some range of *C*_*SUVR*_ (Fig. 2) or SUVR (Fig. 3) where the *FE*_*SUVR*_ trajectory crosses the *x*-axis. At these intercepts, which are cataloged in Tables 1 and 3, the GTM PVC method as applied in this instance is insensitive to errors in the specification of *w*. For most regions, including the global region, the intercepts occur at SUVR below the amyloid positivity threshold (Table 3) and hence a positive error in the GTM width-specification yields a positive correction-factor error at threshold. Exceptions to this are the anterior ventral striatum, insula, and posterior cingulate where the intercept occurs at SUVR greater than the positivity threshold.

Quantitation errors near positivity thresholds are of particular importance. This is addressed in Table 3, which lists *FE*_*SUVR*_ at our GTM-corrected SUVR amyloid positivity thresholds based on the fits for each region. The |*FE*_*SUVR*_| values at the thresholds range from a high of 0.045 mm^−1^ for the orbital frontal to less than 0.01 mm^−1^ for the posterior cingulate. The threshold value of *FE*_*SUVR*_ for the global region is 0.031 mm^−1^.

Because the actual scanner PSF is a function of position within the scanner FOV, a possible concern in using GTM for longitudinal studies is the effect of positioning differences between scans. In the application of GTM, such unaccounted for variation in the true *w* value produces a corresponding effect in the longitudinal-change measures. Given the measured resolution variance as a function of position, described in the “Introduction” section, we expect only sub-millimeter differences in resolution for between-scan positioning errors even up to 5 cm or more, near the radial center of the scanner. If we consider scan-to-scan positioning to result in a typical variation in *w* of 0.5 mm, we would expect typical effects on SUVR to be relatively small, ranging from about 0.004 (posterior cingulate) up to about .023 (orbital frontal) for SUVR in the vicinity of positivity thresholds (Table 3). We note that in this sample, a change in *w* of 0.5 mm would have resulted in no change of global amyloid positivity classification in any of the 29 subjects. For the orbital frontal region, a −0.5 mm change in *w* would result in one subject classification changing from positive to negative, and +0.5 mm change would result in a classification change of one subject from negative to positive.

## Conclusion

The GTM correction, assuming a Gaussian kernel, taken as a function of assumed kernel width, *w*, is reasonably stable. An operating point analysis was performed in which the GTM SUVR error factor *C*_*SUVR*_ was estimated as a linear function of *w* in the vicinity of the assumed operating point (*w* = *w*_0_ = 5 mm (3D)) of our Siemens Biograph mCT. Errors in specification of *w* on the order of 1 mm (3D) mainly produce only modest errors of up to a few percent compared to the overall magnitude of the GTM correction itself. In the global region, operating point SUVR fractional errors per millimeter of *w* error (*FE*_*SUVR*_) ranged from −0.043 to 0.071 mm^−1^ with an RMS value 0.039 mm^−1^. However, some caution is necessary in evaluation of some regions (see Table 2), notably, the anterior ventral striatum which yielded *FE*_*SUVR*_ values ranging from −0.29 to 0.048 mm^−1^. In the vicinity of the University of Pittsburgh positivity thresholds, *FE*_*SUVR*_ values were small in all regions, including global, and ranged from −0.036 to 0.045 mm^−1^.

## Supplementary Information

**Additional file 1: Supplemental Figure 1**. SUV of cerebellar gray matter as a function of GTM FWHM. The SUV scale is logarithmic.

**Additional file 2: Supplemental Table 1**. FreeSurfer 5.3 constituents of the standard [^11^C]PiB quantitation regions. As described in the text, the anterior ventral striatum is composed of regions from the CIC atlas. Cerebellar gray matter is used as a reference region.

**Additional file 3.** Proof that the derivative of the GTM-corrected activity values with respect to w is 0 at w = 0.

## Data Availability

All data directly analyzed during this study are included in this published article. Additional imaging results are available from the corresponding author on reasonable request.
