# Supplementary material for: Characterization of point-spread function specification error on Geometric Transfer Matrix partial volume correction in [11C]PiB amyloid imaging
Source: EJNMMI Phys. 2021 Jul 20;8:54. doi: 10.1186/s40658-021-00403-5 (PMC8292473; doi:10.1186/s40658-021-00403-5)
Supplement: Supplementary file 1 — Additional file 1: Supplemental Figure 1. SUV of cerebellar gray matter as a function of GTM FWHM. The SUV scale is logarithmic. [file 40658_2021_403_MOESM1_ESM.docx]

# Supplemental Material


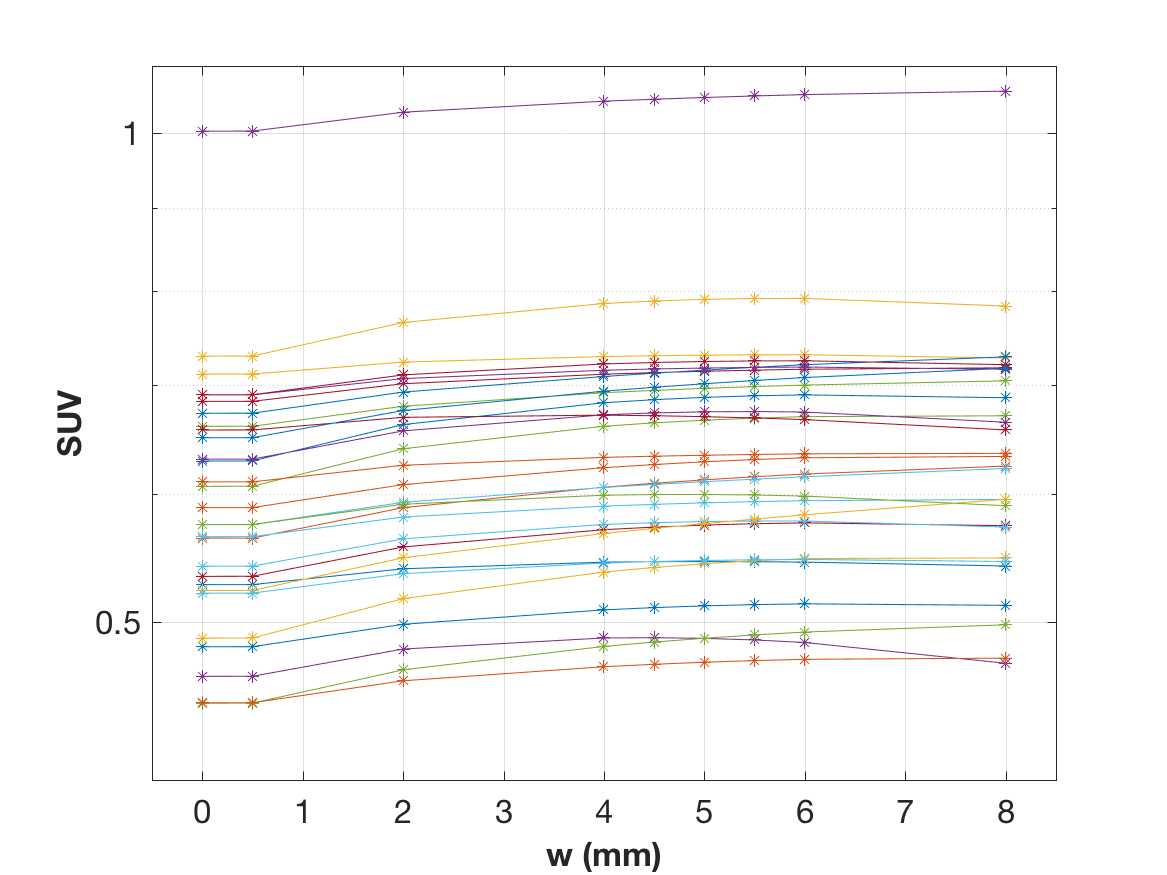


Supplemental Figure 1 – SUV of cerebellar gray matter as a function of GTM FWHM. The SUV scale is logarithmic
