# Supplementary material for: Characterization of point-spread function specification error on Geometric Transfer Matrix partial volume correction in [11C]PiB amyloid imaging
Source: EJNMMI Phys. 2021 Jul 20;8:54. doi: 10.1186/s40658-021-00403-5 (PMC8292473; doi:10.1186/s40658-021-00403-5)
Supplement: Supplementary file 2 — Additional file 2: Supplemental Table 1. FreeSurfer 5.3 constituents of the standard [11C]PiB quantitation regions. As described in the text, the anterior ventral striatum is composed of regions from the CIC atlas. Cerebellar gray matter is used as a reference region. [file 40658_2021_403_MOESM2_ESM.docx]

| Supplemental Table 1 – FreeSurfer 5.3 constituents of the standard [^11^C]PiB quantitation regions. As described in the text, the anterior ventral striatum is composed of regions from the CIC atlas. Cerebellar gray matter is used as a reference region. | | | | | | |
| --- | --- | --- | --- | --- | --- | --- |
| **Composite**  **Quantitation**  **Region** | **FreeSurfer Index** | **FreeSurfer Region** |  | **Composite Region** | **FreeSurfer Index** | **FreeSurfer Region** |
| Anterior Cingulate | 1026 | L Rostral Anterior Cingulate |  | Lateral Temporal Cortex | 1030 | L Superior Temporal |
|  | 1002 | L Caudal Anterior Cingulate |  |  | 1015 | L Middle Temporal |
|  | 2026 | R Rostral Anterior Cingulate |  |  | 1009 | L Inferior Temporal |
|  | 2002 | R Caudal Anterior Cingulate |  |  | 1001 | L Banks of Superior Temporal Sulcus |
|  | | |  |  | 2030 | R Superior Temporal |
| Anterior Ventral Striatum | * | L Ventral Striatum |  |  | 2015 | R Middle Temporal |
|  | * | R Ventral Striatum |  |  | 2009 | R Inferior Temporal |
|  | | |  |  | 2001 | R Banks of Superior Temporal Sulcus |
| Superior Frontal Cortex | 1027 | L Rostral Middle Frontal |  |  | | |
|  | 1003 | L Caudal Middle Frontal |  |  |  |  |
|  | 1028 | L Superior Frontal |  | Parietal Cortex | 1008 | L Inferior Parietal |
|  | 1020 | L Pars Triangularis |  |  | 1029 | L Superior Parietal |
|  | 1032 | L Frontal Pole |  |  | 1031 | L Supramarginal |
|  | 1018 | L Pars Opercularis |  |  | 2008 | R Inferior Parietal |
|  | 1019 | L Pars Orbitalis |  |  | 2029 | R Superior Parietal |
|  | 2027 | R Rostral Middle Frontal |  |  | 2031 | R Supramarginal |
|  | 2003 | R Caudal Middle Frontal |  |  | | |
|  | 2028 | R Superior Frontal |  |  |  |  |
|  | 2020 | R Parstriangularis |  | Posterior Cingulate | 1023 | L Posterior Cingulate |
|  | 2032 | R Frontal Pole |  |  | 1010 | L Isthmus Cingulate |
|  | 2018 | R Pars Opercularis |  |  | 2023 | R Posterior Cingulate |
|  | 2019 | R Pars Orbitalis |  |  | 2010 | R Isthmus Cingulate |
|  | | |  |  |  |  |
| Orbitofrontal Cortex | 1012 | L Lateral Orbitofrontal |  | Precuneus | 1025 | L Precuneus |
|  | 1014 | L Medial Orbitofrontal |  |  | 2025 | R Precuneus |
|  | 2012 | R Lateral Orbitofrontal |  |  | | |
|  | 2014 | R Medial Orbitofrontal |  | Cerebellar Grey Matter | 8 | L Cerebellum Cortex |
|  | | |  |  | 47 | R Cerebellum Cortex |
| Insula | 1035 | L Insula |  |  | | |
|  | 2035 | R Insula |  |  |  |  |
