# Supplementary material for: Characterization of point-spread function specification error on Geometric Transfer Matrix partial volume correction in [11C]PiB amyloid imaging
Source: EJNMMI Phys. 2021 Jul 20;8:54. doi: 10.1186/s40658-021-00403-5 (PMC8292473; doi:10.1186/s40658-021-00403-5)
Supplement: Supplementary file 3 — Additional file 3. Proof that the derivative of the GTM-corrected activity values with respect to w is 0 at w = 0. [file 40658_2021_403_MOESM3_ESM.docx]

Appendix 
Proof that the derivative of the GTM-corrected activity values with respect to w is 0 at w = 0

In Fig. 1 of this article we show plots of apparent SUVR after GTM correction as a function of *w*, the assumed full-width at half-maximum of the scanner point spread function used to perform the GTM correction. It can be seen that the plots have approximately 0 slope at *w* = 0. Here we show that the derivative with respect to *w* of *a*(*w*) is zero at *w* = 0, where *a*(*w*) is the apparent activity concentration after GTM correction using an assumed Gaussian point-spread function of full-width at half-maximum of *w*.

Let m be a one-dimensional array of measured (by PET) activity values, each associated with a particular brain region, e.g., a FreeSurfer region. Let *t* be the one-dimensional array of corresponding true activity values (i.e. uncorrupted by the measurement process). Under the assumption of uniform activity within individual regions and in the absence of stochastic noise, the geometric transfer matrix, *G*, relates these:

$$m=Gt$$

The elements of *G* are determined from the geometry of the various regions of interest and the imaging system matrix, which, as in this case, is typically approximated as a translationally invariant convolution. Based on our model of the GTM kernel (which describes resolution-based mixing of concentrations between regions) as Gaussian, all terms in the matrix elements of *G* that have dependence on *w* have the form:

$f\propto\exp\left[ -\left( \frac{x}{w} \right)^{2} \right]$ (3)

where x is a distance parameter independent of *w*. On a voxel-wise basis, *x* would be proportional to the distance between voxels. On a regional basis, the functional dependencies of matrix-element terms on *w* retain this form since matrix elements on a regional basis are essentially averages over voxel-wise matrix elements. A key point is that the derivatives to all orders of the form *f* with respect to *w*, evaluated at *w* = 0, are equal to zero:

$\left. \frac{\partial^{N}f}{\partial w^{N}} \right|_{w=0}=0$for all integer *N* > 0.

In this article, we investigate the results of misspecification of the smoothing kernel, by varying the assumed value of *w* and performing the GTM procedure for each case. This is achieved by recalculating *G*(*w*) for each *w*-value, and applying the inverse of *G* to *m* to obtain the apparent true activity, *a*(*w*):

$a\left( w \right)=G\left( w \right)^{-1}m$ (4)

The inverse of *G* is given by:

$G^{-1}=\frac{G^{cT}}{\left| G \right|}$ (5)

where *G^cT^* is the cofactor/transpose of *G* and |*G*| is the determinant of *G*. The elements of *m* are constants so the derivative of *a* with respect to *w* is given by:

$\frac{\partial a(w)}{\partial w}=\frac{\partial{G(w)}^{-1}}{\partial w} m$ (6)

From equation (5) we deduce that the terms in the various order derivatives of *G*^-1^ with respect to *w* are all constructed from factors to various powers of

$\frac{1}{\left| G \right|}, G^{cT}, \frac{\partial^{m}\left| G \right|}{\partial w^{m}}, \mathrm{and}\left( \frac{\partial^{l}G^{cT}}{\partial w^{l}} \right)$ (7)

where *k* and *l* are integers. At *w* = 0, *G* is equal to the identity matrix so 1*∕*|*G*(*w* = 0)| = 1. Like *G*, *G^cT^* also reduces to the identity matrix at *w* = 0. However, in any derivatives of *G*(*w*)^-1^, both 1*∕*|*G*| and *G^cT^* will always appear multiplied by some of the derivative factors in (7). With regard to these derivatives - we observe that |*G*| and *G^cT^* both are constructed of terms of factors of the form shown in equation (3). However, products of factors of form (3) maintain this *w*-dependence. Thus, the various order derivatives as well as products of derivatives in (7) equal 0 at *w* = 0. Consequently, derivatives of *G*^-1^ to all orders with respect to *w* and therefore derivatives to all orders of *a* (equation 4) vanish at *w* = 0.
